# Clinicopathologic characteristics of severe COVID-19 patients in Mexico City: A post-mortem analysis using a minimally invasive autopsy approach

**DOI:** 10.1371/journal.pone.0262783

**Published:** 2022-03-03

**Authors:** Carlos Nava-Santana, María Rodríguez-Armida, José Víctor Jiménez, Nancy Vargas-Parra, Diana E. Aguilar León, Alejandro Campos-Murguia, Ricardo Macías-Rodriguez, Andrés Arteaga-Garrido, Antonio C. Hernández-Villegas, Guillermo Dominguez-Cherit, Eduardo Rivero-Sigarroa, Armando Gamboa-Dominguez, Alfonso Gullias-Herrero, José Sifuentes-Osornio, Norma Ofelia Uribe-Uribe, Luis E. Morales-Buenrostro

**Affiliations:** 1 Department of Nephrology, Instituto Nacional de Ciencias Médicas y Nutrición Salvador Zubirán, Mexico City, México; 2 Department of Medicine, Instituto Nacional de Ciencias Médicas y Nutrición Salvador Zubirán, Mexico City, México; 3 Department of Pathology, Instituto Nacional de Ciencias Médicas y Nutrición Salvador Zubirán, Mexico City, México; 4 Department of Gastroenterology, Instituto Nacional de Ciencias Médicas y Nutrición Salvador Zubirán, Mexico City, México; 5 Department of Radiology, Instituto Nacional de Ciencias Médicas y Nutrición Salvador Zubirán, Mexico City, México; 6 Department of Critical Care Medicine, Instituto Nacional de Ciencias Médicas y Nutrición Salvador Zubirán, Mexico City, México; National Institutes of Health, UNITED STATES

## Abstract

**Objective:**

Describe the histological findings of minimally ultrasound-guided invasive autopsies in deceased patients with severe SARS-CoV-2 and compare the diagnostic yield with open autopsies.

**Design:**

Observational post-mortem cohort study. Minimally invasive ultrasound-guided autopsies were performed in fourteen deceased patients with a confirmed diagnosis of SARS-CoV-2 pneumonia. Histological and clinical findings of lung, kidney, and liver tissue are described and contrasted with those previously reported in the literature.

**Setting:**

Single-center COVID-19 reference center in Mexico City.

**Results:**

Fourteen minimally invasive autopsies revealed a gross correlation with open autopsies reports: 1) Lung histology was characterized mainly by early diffuse alveolar damage (12/13). Despite low lung compliances and prolonged mechanical ventilation, the fibrotic phase was rarely observed (2/13). 2) Kidney histopathology demonstrated acute tubular injury (12/13), interstitial nephritis (11/13), and glomerulitis (11/13) as the predominant features 3) Liver histology was characterized by neutrophilic inflammation in all of the cases, as well as hepatic necrosis (8/14) despite minimal alterations in liver function testing. Hepatic steatosis was observed in most cases (12/14). SARS-CoV-2 positivity was widely observed throughout the immunohistochemical analysis. However, endothelitis and micro thrombosis, two of the hallmark features of the disease, were not observed.

**Conclusion:**

Our data represents the largest minimally invasive, ultrasound-guided autopsy report. We demonstrate a gross histological correlation with large open autopsy cohorts. However, this approach might overlook major histologic features of the disease, such as endothelitis and micro-thrombosis. Whether this represents sampling bias is unclear.

## Introduction

Since the emergence and spread of the SARS-CoV-2 virus epidemic, unraveling this new disease’s pathophysiological mechanisms through post-mortem histological studies has become a growing need [1, 2]. Large postmortem cohorts have demonstrated diffuse alveolar damage (DAD) as the predominant histologic feature of this disease [3–6]. A phenotype consistent with acute respiratory distress syndrome (ARDS) similar to other viral causes such as MERS or SARS [7]. Clinical evidence of kidney involvement has been reported in up to 40% of cases, with acute tubular injury or necrosis (ATI) being the most common histological pattern [8, 9]. Nonetheless, several additional reports have added key observations that provide a complete view of the disease. Varga et al. [10] made the initial description of multiorgan endotheliitis (including the lung) that was later supported by a detailed analysis of endothelial inflammation and the expression of molecular markers of angiogenesis and immunothrombosis [11], which strongly suggested the capillary endothelium as a central mediator of damage in COVID-19 [12].

Although multiple cohorts and case series have reported the histological findings of open autopsies, only a few cases have described the use of minimally invasive autopsies (MIA) or percutaneous biopsies [13–15]. MIA is a percutaneous, needle-based approach for tissue collection without opening body cavities. This approach has been validated and protocolized in low-income countries, particularly in the setting of biohazard concerns and highly contagious diseases (tuberculosis, yellow fever, and Nipah virus) [15–19]. Even though complete open autopsy remains the gold standard for multi-organic post-mortem diagnosis, the unique challenges posed by the COVID-19 pandemic regarding biosafety precautions (particularly in low-income countries) made this technique unavailable [20]. An ultrasound-guided approach for MIA (MIA-US) has been described yielding similar findings to those reported in open autopsies. However, the efficiency for tissue obtention was low overall (5/60 MIAs were viable for analysis) [21]. Recently, Rakislova et al. compared the performance of protocolized MIAs with matched full-body open autopsies in the same patients. Although the sample was small (five patients), their results revealed a similar diagnostic yield [22]. A report from Belgium showed that this technique led to changes in the diagnosis of death in 15/18 of their COVID-19 deceased patients [23]. The main advantage of a MIA-US rely on the feasibility of performing bedside tissue sampling without requiring transportation to an autopsy room, an therefore reducing the exposed personnel.

Herein, we present the histopathological findings of MIA-US in fourteen deceased patients with SARS-CoV-2 confirmed infection who died in a COVID-19 referral center in Mexico City. We aim to describe and compare our findings to those widely known in large cohorts of open autopsies. In addition, we make a detailed description of the patients’ clinical, radiological, and histological findings.

## Methods

### Study design

An observational study including 14 consecutive deceased patients with a confirmed diagnosis of SARS-CoV-2 infection at Instituto Nacional de Nutrición y Ciencias Médicas “Salvador Zubirán”; a tertiary care center in Mexico. The diagnosis was confirmed with a positive real-time polymerase chain reaction [24]. The patient’s electronic medical records were reviewed to obtain the demographic data, clinical features, and laboratory findings.

#### Ethics committee approval

The study received ethical approval from the Ethics and Research Committee of the Instituto Nacional de Nutrición y CIencias Médicas “Salvador Zubirán” (IRB approval number 3357). Written informed consent was obtained from the patient’s next of kin after a detailed explanation of the study and procedures performed. An information sheet containing the contact details of the research team was delivered.

#### Tissue obtention and ultrasonographic technique

After written informed consent was obtained, postmortem biopsies of the kidney, lung, and liver were obtained within 3 hours of death. All medical staff involved in tissue sampling and processing were equipped with the appropriate personal protection equipment throughout all aerosol-generating procedures. Instruments and supplies were disinfected or discarded accordingly with the hospital´s central sterile supply department (CSSD) protocols and international sanitary recommendations [25].

Postmortem biopsies were performed bedside (the same bed were the patient passed), using a 16-gauge core needle anatomically guided with a Sonosite X-Porte ultrasound (SonoSite X-Porte; Fujifilm, Tokyo, Japan). Pre-mortem diagnostic imaging was reviewed for gross anatomic localization and bedside scanning was performed by two POCUS proficient physicians (CNS, MRA) and an interventional radiologist (AHV) for anatomic localization.

For lung biopsies a HFL38xp lineal 13–6 MHz transducer (SonoSite X-Porte; Fujifilm, Tokyo, Japan) was used. Under direct visualization, the needle tip was inserted and followed along the soft tissue structures and pleural surface. Both right and left lungs mid-clavicular samples were obtained, the needle was advanced 2–5 cm depending on the fat tissue of the subject. This procedure was repeated 2–3 times in each lung, each puncture was performed with a minimal 1 cm distance from the previous needle pass in order to avoid biased sampling. For liver biopsies a right lateral view was obtained targeting the right lobe (AHV analyzed each image before tissue acquisition) a total of 3–4 needle passes was performed with a minimal 1 cm distance from the previous puncture. For renal biopsies the patient was placed in prone position for adequate kidney visualization. The needle was followed through the soft tissue structures avoiding the liver parenchyma until reaching the renal cortex. A total of 2–3 needle passes per kidney was performed with a minimal 1 cm distance from the previous puncture. For both liver and renal biopsies a C35xp convex 8–3 MHz transducer (SonoSite X-Porte; Fujifilm, Tokyo, Japan) was used.

Our research protocol pre-specified the acquisition of 7–10 samples for each tissue with a total of 21–30 specimens per subject, the specimen size was considered satisfactory for analysis with 5–10 mm^2^ of tissue.

Tissue samples were immediately fixed with 10% neutral-buffered formalin for at least 24 hours for viral inactivation and sample preservation.

#### Tissue preparation and analysis

All tissue samples were processed under standard BSL-2 (biosafety laboratory) measures in the Department of Pathology. Tissue samples were paraffin-embedded, sectioned, stained with hematoxylin and eosin (H&E), periodic acid of Schiff (PAS), Masson’s trichrome stain, and Methenamine silver satin. A renal pathologist (NU) and a general pathologist (NV and DA) analyzed all biopsies, light microscopy, and immunohistochemistry. Owed to the similarity of the subtle alterations found in renal biopsies, we decide to use operational definitions correspondent to those described in Banff Classification for kidney transplant [26]. Mild glomerulitis: intracapillary margination of inflammatory cells in <25% of glomeruli, moderate: 25–75%, severe: >75% of glomeruli. Mild interstitial inflammation in 10–25% of total cortical parenchyma inflamed, moderate in 26–50%, severe in >50%. Mild tubular atrophy involving up to 25% of the area of cortical tubules, moderate involving 26–50%, severe involving in >50%. Mild Interstitial fibrosis: in 6–25% of cortical area, moderate in 26–50%, severe in >50% of cortical area. In order to grade histological liver lesions, activity score for histological classification of nonalcoholic steatohepatitis was used [27]. Mild steatosis: 5%–33% affected hepatocytes, moderate: 34%–66%, severe: >66%. Mild neutrophilic sinusoidal inflammation: <2/20x, severe: ≥2/20x. Other histological characteristics were evaluated as binomial variables.

*Immunohistochemistry*. Deparaffinized 2-μm sections were used for immunohistochemical analysis for COVID-19 and CD68. Briefly, antigen retrieval was performed by immersing the slides in ANTIGEN RETRIEVAL BUFFER (DIVA DECLOAKER, BIOCARE MEDICAL DV2200SC23), for twenty-five minutes in boiling water. Endogenous peroxidase activity was inhibited by immersing the slides in PEROXIDE BLOCK (CELL MARQUE CMC925660030) 20 min, the slides were incubated 2 HR at room temperature with antibodies against SARS-CoV-2 1:250 (SARS-CoV-2 spike antibody 1a9, Gene Tex. The slides were washed five times in PBS 1X, pH 7.4, incubated slide with HI-DEF DETECTION HRP polymer system (CELL MARQUE CMC954080040) 30 minutes at room temperature, and revealed with dab substrate (CELL MARQUE CMC957080031) incubate until desired color (10 mins). The reaction was arrested with water, add second antibody CD68 (KP1)(CM 033ABC 1:100 DILUTION), incubate slide 40 min at room temperature, washed five times in PBS 1X pH7.4 incubate slide with HE DER DETECTION ALK PHOS POLYMER SYSTEMS (CELL MARQUE 962D-30) and revealed with RED CHROMOGEN KIT (CELL MARQUE 956d). The slides were counterstained with hematoxylin. Thereafter, the tissues were washed in distilled water for five minutes, dehydrated sequentially in 70%, 90% and 100% ethanol, ethanol/xylene and xylene, and mounting resin.

#### Patient and public involvement

Patients and the public were not involved in the design, conduct or dissemination of this observational study.

## Results

Between April 1^st^ and July 1^st^, 2020, 14 patients were included in the study. The median age was 54 years (IQR 47–62), 78% were men. The mean duration of hospitalization until the fatal outcome was 19 days (IQR 8.5–26). All of the patients developed severe ARDS and required invasive mechanical ventilation.

At least one comorbidity was found in all patients, obesity in eight (57%), overweight in five (35%), and only one with a normal BMI. Six (43%) patients had type 2 diabetes mellitus (DMT2), two (14%) hypertension, and two more with an underlying malignancy (14%).

Baseline patient characteristics are described in Table 1 and the final (last values available before death) laboratory values and severity markers of COVID-19 in Tables 1 and 2.

**Table 1:** Patient characteristics.

Patient	Gender	Age	BMI	Comorbidities	Smoking history	Baseline eGFR (mL/min/1.73 m^2^)	AKI/ RRT	LOS (days)
1	M	48	35	Obes, T2DM, HTN	No	104	No/No	5
2	M	49	34.6	Obes	No	52	Yes/No	7
3	M	37	34.1	Obes, T2DM	No	101	No/No	7
4	M	59	31.2	Obes, T2DM, IHD	Yes	40	Yes/Yes	29
5	F	51	33.9	Obes	No	51	Yes/No	25
6	M	50	28.1	Cardiac valve replacement	Yes	100	Yes/No	9
7	F	41	44.4	Obes	No	108	Yes/No	12
8	M	48	31.4	Obes, T2DM	Yes	94	Yes/No	20
9	M	56	27	T2DM	No	83	Yes/No	23
10	M	43	40.5	Obesity	No	99	Yes/Yes	16
11	M	70	26.6	Prostate cancer	Yes	91	No/No	31
12	M	53	28.3	T2D	Yes	73	Yes/No	29
13	F	70	24.4	MDS	No	81	No/No	38
14	M	76	26.2	HTN	Yes	83	Yes/No	24

BMI: body mass index LOS: length of stay, AKI: acute kidney injury, RRT: renal replacement therapy, eGFR: estimated glomerular filtration rate. MDS: myelodysplastic syndrome. Obes: obesity, T2DM: type 2 diabetes mellitus, HTN: hypertension, IHD: ischemic heart disease.

**Table 2:** COVID-19 severity markers at time of death.

Patient	PaO_2_/ FiO_2_	ALC (cell/ μL)	LDH (U/L)	DD (ng/mL FEU)	Fg (mg/dL))	Ferritin (ng/mL)	CPK (U/L)	TG (mg/dL)	CPR (mg/dL)
1	88	1335	252	NA	NA	1078	NA	NA	33.5
2	173	612	709	1010	574	2128	2471	358	3.3
3	34	798	364	3164	508	439	364	190	20.4
4	283	825	472	1824	465	319	135	283	4.6
5	104	732	557	4457	379	1634	147	156	2.3
6	195	1186	668	1189	1000	1342	3301	337	20.6
7	228	2072	299	1414	1000	279	232	391	15.9
8	127	664	310	2423	981	1483	854	211	21.6
9	59	787	303	1468	328	1367	286	306	31.7
10	54	775	592	2038	NA	4404	1863	355	2.1
11	68	936	358	703	482	396	399	206	6
12	167	1092	386	1975	734	651	324	156	10.4
13	119	3210	282	852	597	1471	128	428	13
14	102	1212	352	1669	460	838	373	178	9.8

ALC: absolute lymphocyte count, LDH: lactate dehydrogenase, DD: D-dimer, Fg: fibrinogen, creatine phosphokinase, TG: triglyceride, CRP: C-reactive protein.

### Lung pathology

**Fig 1:**
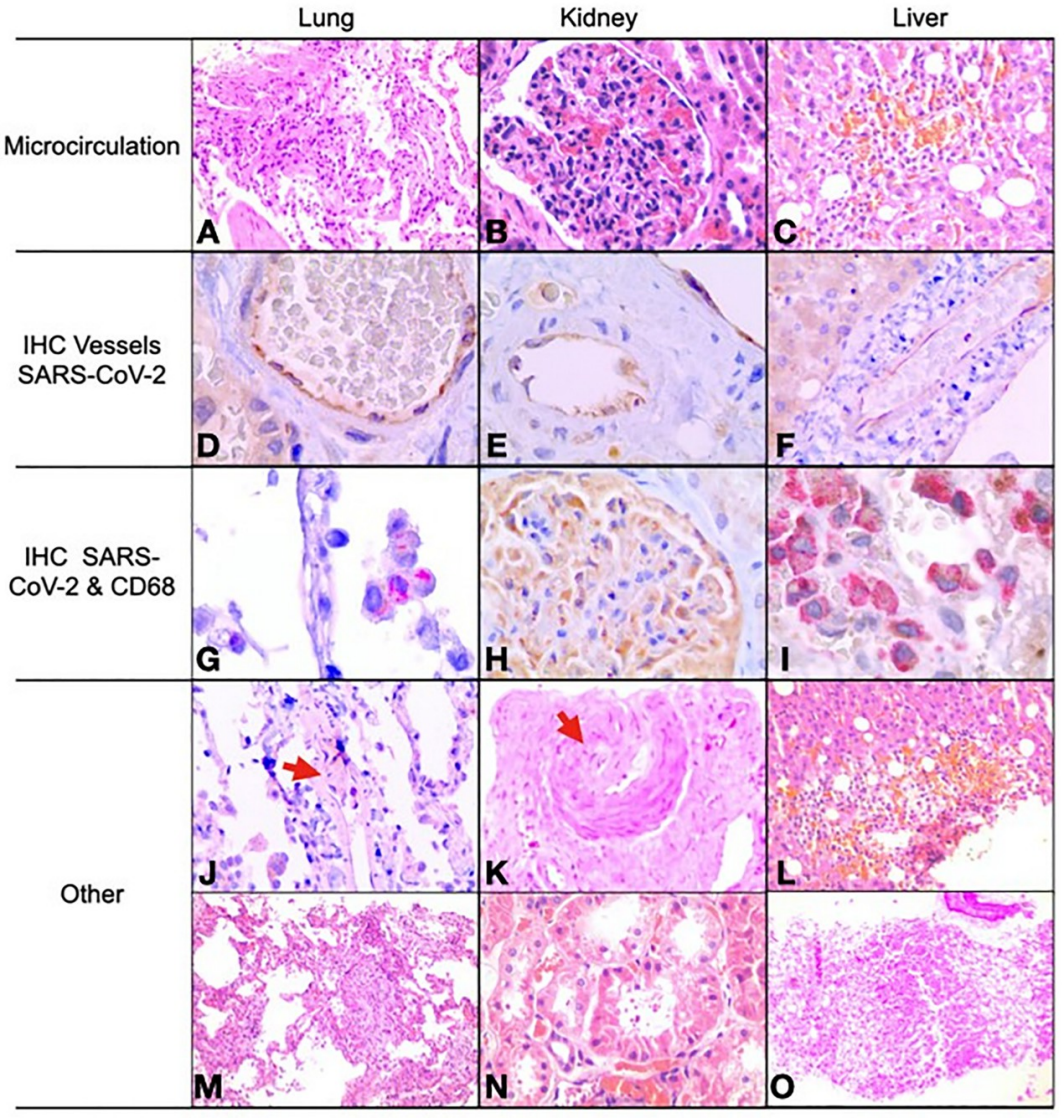
Histological and immunohistochemical findings. Microcirculation damage **(A, B, C): A**. Diffuse alveolar damage in exudative phase (H-E, 40X) **B**. Glomerulitis (H-E, 40X); **C**. Lobular hepatitis (H-E, 20X); Sars-Cov-2 in endothelial cells by immunohistochemistry **(D, E, F): D**. Anti-Sars-Cov2 cytoplasmic positivity in endothelial cells of pulmonary arterial vessels, 100X **E**. In endothelial cells of kidney arterial vessel, 100X and **F**. In endothelial cells of the hepatic arterial vessel, 40X. Sars-Cov-2 present in other cells (IHC) **(G, H, I): G**. Cytoplasmic positivity in intra alveolar macrophages with colocalization of anti-Sars-Cov2 and CD68, 100X; **H**. Visceral epithelial cells (podocytes) and glomerular parietal cells for anti-Sars-Cov2, 100X. **I**. Colocalization with anti-Sars-Cov2 (tobacco brown) and CD68 (red) in Kupffer cells, 100X; Other findings **(J, K, L, M, N, O): J**. Intraluminal organized thrombus in pulmonary arteriole, 40X **K**. Acute focal thrombotic microangiopathy in renal tissue (H-E, 10X); **L**. Hepatocellular hemorrhagic necrosis (H-E, 20X); **M**. Diffuse alveolar damage in proliferative phase (H-E, 40X); **N**. Cytoplasmic isometric vacuolization of renal tubular epithelium (H-E, 60X); **O**. Macrovesicular hepatic steatosis (H-E, 4X).

Thirteen lung biopsies were included in the analysis, tissue samples for one patient was not viable for tissue processing and analysis. In twelve (92%) of the cases, the histological examination revealed diffuse alveolar damage (DAD) in different stages of the disease (three on exudative phase, seven proliferative, two fibrotic), all of these samples had congestive capillaries, and eight showed signs of hemorrhage in the light microscopy. Nine of the 13 cases had interstitial inflammation, primarily heterogeneous; additionally, three revealed medial hyperplasia as a marker of pulmonary arterial hypertension. Only one showed signs of necrosis and thrombosis, and one more bronchiolitis obliterans with organizing pneumonia (BOOP) as the main pathologic finding. None of the cases showed endothelitis or microthrombi. IHC for SARS-Cov-2 detection revealed cytoplasmic positivity, mainly in endothelial cells (11/13) (Fig 1F), followed by macrophages (10/13) (Fig 1I), pneumocytes (5/13), desquamated cells (3/13), and arterial smooth muscle cells in 2 cases. Interstitial inflammation was noted in 9 cases, of which five corresponded to mixed lymphoplasmacytic and neutrophilic infiltration, two of mixed type with predominance of eosinophils, and 2 of lymphoplasmacytic type. A more detailed description of lung pathology is found in Table 3.

**Table 3:** Lung biopsy findings.

Summary of lung histopathologic findings	
Diffuse Alveolar Damage	92% (12/13)
- Exudative Phase	23% (3/13)
- Proliferative Phase	54% (7/13)
- Fibrotic Phase	15% (2/13)
Bronchiolitis obliterans with organizing pneumonia (BOOP)	7% (1/13)
Vascular Findings	
- Medial Hyperplasia	23% (3/13)
- Endotheliitis	0% (0/13)
- Microthrombi	0% (0/13)
- Macrothrombi	7% (1/13)
Interstitial Inflammation	
- Mixed	69% (9/13)
- Mixed- Eosinophil	56% 5/9
Predominant	22% 2/9
- Lympho-plasmacytic	22% 2/9
Pleuritis	7% (1/13)
Capillary Congestion	100% (13/13)

#### Lung clinical/radiological characteristics

Seven of the 14 patients initially presented with severe ARDS (Pa/FiO2 ratio < 150 mmHg), but all of them developed severe ARDS requiring invasive mechanical ventilation during their hospital stay, and 13/14 presented severe involvement in chest CT scan evaluation (>50% involvement). They received standard of care for ARDS, mechanical ventilation under lung-protective strategies as recommended by ARDSNet protocol. All of them received prone positioning due to severe hypoxemia. Regarding the pathophysiologic parameters of lung mechanics: all of the patients had reduced static compliance of the respiratory system presenting with a mean of 28 ml/cmH2O and further deteriorating to a mean of 24 ml/cmH2O before death. Only four of the 14 patients (28.6%) received mechanical ventilation for less than ten days, five during 10–20 days, and five more than 20 days. See Table 4 for further detail.

**Table 4:** Physiological and lung mechanical characteristics.

Summary of Physiological and Lung Mechanical Characteristics	
Severe ARDS	100% 14/14
Treatment	
- Lung Protective Ventilation (ARDSNET)	100% (14/14)
- Prone Positioning	100% (14/14)
Days on IMV	
- <7 Days	28% (4/14)
- 7–15 Days	22% (3/14)
- > 15 Days	50% (7/14)
Initial Static Compliance (ml/cmH2O)	
- >40	14% (2/14)
- 20–40	86% (12/14)
- < 20	0% (0/14)
Final Static Compliance (ml/cmH2O)	
- >40	7% (1/14)
- 20–40	75% (9/14)
- < 20	28% (4/14)
% Lung involvement in CT Scan	
- Mild	0% (0/14)
- Moderate	7% (1/14)
- Severe	93% (13/14)

### Renal histopathology and renal function

Thirteen kidney biopsies were suitable for histological analysis. The most common finding was ATI in 12 patients (92%), glomerulitis (of variable degrees) in 11 (84%), eight of them showed moderate to severe lesions. Focal thrombotic microangiopathy was seen only in one patient. Other findings, such as diabetic nephropathy, were seen in five patients (38%). Immunohistochemistry (IHC) reaction against Sars-Cov2 showed cytoplasmic granular positivity in endothelial cells of arteries, arterioles, vasa recta, and veins in all cases (100%) (Fig 1D). Also, all cases displayed granular cytoplasmic positivity in glomerular epithelial cells (visceral and parietal) (Fig 1G), A more detailed description of the pathological descriptions can be found in Table 5. Noteworthy, 10 of the 14 patients (71%) developed AKI during the ICU stay, yet only two of them (14%) required some type of renal replacement therapy (RRT). Immunohistochemical (IHC) assay for detection of SARS-CoV-2 was positive predominantly in glomerular epithelial and endothelial cells in all the patients.

**Table 5:** Renal biopsy findings.

Summary of Renal Biopsy Findings	
Acute Tubular Injury	92% (12/13)
Glomerulitis	84% (11/13)
- Mild	36% (4/11)
- Moderate	36% (4/11)
- Severe	28% (3/11)
Interstitial Nephritis*	77% (10/13)
- Mild	100% (10/10)
Regenerative Changes	38% (5/13)
Vacuolar degeneration	23% (3/13)
Global/Segmental Glomerulosclerosis	61% (8/13)
Tubular Atrophy	
- Mild (<25%)	92% (12/13)
- Moderate (26–50%)	92% (11/12)
- Severe (>50%)	8% (1/12)
Positive SARS-CoV-2 (IHC)	100% (13/13)
(Granular cytoplasmic staining)	100% (13/13)
Glomerular epithelial cells	61% (8/13)
Tubular epithelial cells	61% (8/13)
Endothelial cells	84% (11/13)
Nervorum	8% (1/13)
Urothelial cells	8% (1/13)

*All of the cases marked as interstitial nephritis had an infiltrate of 5% or less.

### Liver histopathology and biochemistry

Fourteen liver biopsies were suitable for histopathological analysis. Neutrophilic sinusoidal inflammation was present in all patients, and IHC for CD68 revealed increased interstitial macrophages. Macrovesicular steatosis was found in 12 (86%), ballooning degeneration of hepatocytes in seven (50%), and lobular inflammation in nine (64%). Two patients had advanced liver fibrosis, one F3 and one F4. Immunohistochemical assay (ICH) for detection of SARS-CoV-2 was positive in 12 biopsies (86%), 11 (79%) in the endothelial cells, eight (57%) in macrophages, and one showed positivity around hepatocytes. Concerning liver biochemistry, seven patients (50%) had levels above the upper limit of normal (ULN). Six patients (43%) had increased alkaline phosphatase, and none had increased bilirubin at admission. A more detailed description of liver pathology can be found in Table 6.

**Table 6:** Liver biopsy findings.

Summary of liver histopathologic findings	
Liver steatosis	86% (12/14)
- Mild	57% (8/14)
- Moderate	14% (2/14)
- Severe	14% (2/14)
Lobular inflammation	64% (9/14)
Hepatocyte ballooning	50% (7/14)
Advanced liver fibrosis	14% (2/14)
Neutrophilic sinusoidal inflammation	100% (14/14)
- Mild	29% (4/14)
- Severe	71% (10/14)
Necrosis	57% (8/14)
Cholestasis	50% (7/14)
Positive SARS-CoV-2 (IHC)	86% (12/14)
Macrophages	57% (8/14)
Endothelial cells	78% (11/14)
Hepatocytes	7% (1/14)

### Treatment

The patients reported in this cohort did not receive dexamethasone or its equivalent. All the patients in this study were enrolled before the RECOVERY preliminary report. All of the patients received prophylactic weight-adjusted anticoagulation. None of the patients received biologics or experimental drugs.

## Discussion

To our knowledge, we report the largest series of MIA-US focusing on lung, kidney, and liver histology in COVID-19 deceased patients. Our findings revealed similar gross histological findings to those reported in open autopsies cohorts. However, pulmonary hallmark findings commonly reported in COVID-19 pathology (micro thrombosis and endothelitis) were not observed in our study.

The main histologic pulmonar finding in our series was DAD, especially in the exudative and proliferative phases (hyaline membrane formation and atypical pneumocyte hyperplasia), which is consistent with large autopsy cohorts [3–5, 28]. Small case series have reported a higher incidence of fibrotic phases [29]. However, this finding was not observed in this study, even though most patients had prolonged mechanical ventilation and low lung compliances.

These findings are similar to those observed in ARDS induced by other viruses such as MERS or SARS [7]. However, a direct histological comparison using controls (from autopsies affected by other viral causes of pneumonia) is lacking. An indirect estimate was reported by Hariri et al., who elaborated a systematic review of the literature compared autopsy findings of COVID-19 with influenza H1N1 and SARS ARDS. Their results suggest a similar frequency of DAD. Interestingly a higher frequency of micro thrombosis was observed in SARS (58%) and COVID-19 (57%) compared with influenza (28%) [30], suggesting a particular propensity of coronaviruses towards endothelial damage and micro thrombosis [31, 32]. The central role of endothelial capillary damage in COVID-19 pathogenesis has been provocatively suggested by Vargas et al. [10] and later supported by analysis of molecular markers associated with angiogenesis [11]. However, our study did not find evidence of endothelitis or micro thrombosis.

Whether the lack of pulmonar endothelitis and micro thrombosis in our study represents sampling bias or not is unclear. Although previous open autopsy cohorts have described these characteristics as a frequent feature of the disease [3, 5, 33], a large (100 autopsies) cohort failed to replicate these findings. Interestingly this contrasts with a recent systematic review where 123/263 lung biopsies (47%) presented with microthrombi and 44/263 (17%) with endothelial inflammation resembling vasculitis [34]. To be noted, these studies only included ten MIA in their analysis.

The few MIA reports in the literature have shown mixed results as well. Early case series from Brazil and China showed microthrombi in most (8/10 and 9/10, respectively) of their MIA samples [14, 34]. However, further reports have failed to confirm these findings (only 2/16) [13, 14, 19, 35]. This variability within open autopsies and MIA is similar. Whether it represents the intrinsic heterogeneity of the disease or a pattern of decreased inflammation along time attributable to anti-inflammatory treatment (corticosteroids, tocilizumab) is unclear. However, most of the cohorts and case series were finalized either before the RECOVERY trial preliminary report was released or within weeks it happened [36]. The inherent susceptibility for sampling bias in MIA and MIA-US further complicated the interpretation.

Macrothrombosis was rare despite elevated D-dimer levels, however patients underwent imaging (angiotomography) when a clinical suspicion of pulmonary thromboembolism was deemed reasonable, not routinely. Therefore, underdiagnosis of macrothrombosis (particularly in the setting of medial hyperplasia in the pulmonary arteries) is possible. The particular phenotype of low lung compliances, elevated D-dimer, and absence of macro thrombotic events foresees a dismal prognosis, as reported in a large observational study of lung mechanics [30]. The absence of micro thrombosis was a striking finding in our study. All of our patients received anticoagulation during their hospital stay, and none of them was diagnosed with pulmonary embolism or any other thrombotic event.

ATI was present in most patients regardless of overt acute kidney injury (AKI), consistent with previous reports [37–39]. Despite profound elevation in serum creatinine (AKI stage 2–3), ATI was mild in most cases, which the multifactorial nature of AKI in COVID-19 might explain. Out of the two patients that required RRT, one had ATI and severe glomerulitis, while the other was found with mild ATI. We found no evidence of acute interstitial nephritis (AIN).

The most striking findings in the present series correspond to glomerulitis (more significant than tubulointerstitial inflammation) and endothelial involvement which may reflect acute endothelial inflammation of the kidney microvasculature [40, 41]. Almost all cases had positive IHC for SARS-Cov-2 in visceral and parietal epithelial glomerular cells, which have been inconsistently reported in the literature and subject to controversy [37, 38]. Although other forms of glomerular pathology have been described, none of them was observed in this cohort. Primary pathology of preexisting conditions was also observed [42].

The most common liver findings were neutrophilic sinusoidal inflammation (14/14), macrovesicular steatosis (12/14), and a positive SARS-CoV-2 IHC in 12/14 biopsies. Liver injury mediated by neutrophils is found in various pathologies, DILI, viral hepatitis, and liver steatosis [43], all of these possible mechanisms of liver damage in patients with COVID-19. Neutrophil accumulation and acute inflammation often cause collateral damage in the liver tissue of patients with viral infections [44]. SARS-CoV-2 particles have been found in hepatic tissue in 50–100% of COVID-19 patients [45, 46]. The presence of marked DNA traps, early NET formation, and NET-platelets aggregates have been demonstrated and hypothesized as immune thrombogenic [47]. In this series of cases, 78% of the patients had positive SARS-CoV-2 IHC in endothelial cells (and only 7% in hepatocytes), suggesting that neutrophils could directly respond to the viral load in liver tissue.

Macrovesicular steatosis was a common feature in this series, present in 12/14 patients, with a high proportion of our patients being overweight and obese. The latter is consistent with previous post-mortem reports where macrovesicular steatosis was the most common finding (50–75% of the patients) [46, 48]. It is noteworthy that a considerable proportion (2/14) of the cohort had advanced liver fibrosis. This finding has been described similarly in a large cohort of autopsies at Mount Sinai [4]. In this same cohort, outflow obstruction and early organizing thrombi were noted in up to 60% (62/92) of the cases. These findings were not replicated in this study. Lastly, despite the fact that MIA-US do not provide a perfect correlation with open autopsies it does offer a bedside alternative for tissue sampling in low resource setting where autopsy rooms are not available or limited.

## Conclusions

In this study, we analyzed minimally invasive biopsies to characterize and correlate the histological findings of deceased patients with severe COVID-19 ARDS with those previously described in open, conventional autopsies. Our findings broadly correlate with those previously reported in lung, kidney, and liver post-mortem autopsies. However, pulmonar endothelitis and micro thrombosis, two hallmark features of this disease, were rare findings in this study. Nevertheless, DAD was present as a consequence of lung microvasculature involvement. Rather than a particular characteristic of our population, the absence of these features might be secondary to sampling bias. The latter represents a considerable limitation of this minimally invasive approach, particularly when unraveling the pathophysiological implications of tissue analysis.

Another limitation of this study is the lack of in situ hybridization or in situ RT-PCR for viral detection, which would provide more information about the pathophysiology of the disease. Although we did not have matched controls for optimal comparison, previous reports have shown a good correlation between both methods [22]. Overall this study supports the use of MIA-US in resource-limited settings for the gross diagnostic characterization of this disease, providing a good oversight of pathologic tissue abnormalities. However, it might not be the optimal method for settling the physiopathological controversies of the disease.

## Supporting information

S1 File (DOCX)
