# Supplementary material for: Clinicopathologic characteristics of severe COVID-19 patients in Mexico City: A post-mortem analysis using a minimally invasive autopsy approach
Source: PLoS One. 2022 Mar 3;17(3):e0262783. doi: 10.1371/journal.pone.0262783 (PMC8893646; doi:10.1371/journal.pone.0262783)
Supplement: S1 File — (DOCX) [file pone.0262783.s001.docx]

**Table S1. Baseline laboratory values.**

| Patient | HGB  (g/dL) | WBC  (x10^3/μL) | PLT  (K/ μL) | SCr  (mg/dL) | TBIL  (mg/dL) | ALT  (U/L) | AST  (U/L) | ALP  (U/L9 | Alb  (g/dL) | INR | PTT  (seg) |
| --- | --- | --- | --- | --- | --- | --- | --- | --- | --- | --- | --- |
| 1 | 17.2 | 11.7 | 279 | 0.8 | 0.6 | 23 | 20 | 84 | 4.9 | 1 | 30.5 |
| 2 | 16.9 | 16.7 | 342 | 1.5 | 1.5 | 1598 | 3175 | 74 | 3.2 | 1.2 | 35.7 |
| 3 | 15.8 | 15 | 225 | 0.9 | 0.5 | 60 | 49 | 153 | 2.6 | 1.2 | 25.1 |
| 4 | 9 | 10.4 | 451 | 1.8 | 0.3 | 20 | 47 | 81 | 4 | 1.2 | 39.9 |
| 5 | 14.7 | 32 | 367 | 1.2 | 0.4 | 47 | 45 | 140 | 3.2 | 1 | 31.1 |
| 6 | 17.3 | 5.4 | 171 | 0.8 | 0.8 | 222 | 141 | 111 | 4.4 | 1 | 32.1 |
| 7 | 13.6 | 12.7 | 301 | 0.7 | 0.4 | 104 | 112 | 113 | 3.2 | 1 | 23.9 |
| 8 | 17.4 | 11.4 | 188 | 0.9 | 0.9 | 22 | 17 | 94 | 4.1 | 1.2 | 27.9 |
| 9 | 16.7 | 3.7 | 180 | 1 | 0.8 | 57 | 80 | 72 | 4.2 | 1 | 29.4 |
| 10 | 17.3 | 20.4 | 304 | 0.9 | 0.8 | 28 | 32 | 77 | 3.8 | 1.2 | 26 |
| 11 | 15.9 | 10-7 | 222 | 0.7 | 1 | 29 | 55 | 79 | 3.2 | 1.2 | 28.1 |
| 12 | 14.9 | 11.4 | 283 | 1.1 | 0.6 | 25 | 40 | 194 | 3 | 1.1 | 26 |
| 13 | 12.3 | 12.9 | 187 | 0.7 | 1.9 | 52 | 32 | 173 | 3.8 | 1.3 | 63.8 |
| 14 | 15.5 | 17.2 | 257 | 0.8 | 0.6 | 20 | 57 | 79 | 2.8 | 1.1 | 33.1 |

*HGB: hemoglobin, WBC: white blood cell, PLT: platelet, SCr: creatinine, TBIL: total bilirubin, ALT: alanine aminotransferase, AST: aspartate aminotransferase, Alb: albumin, INR: international normalized ratio, PTT: partial thromboplastin time.*

**Table S2. COVID-19 severity markers at baseline.**

| Patient | PaO_2_/  FiO_2_ | ALC  (cell/ μL) | LDH  (U/L) | DD  (ng/mL FEU) | Fg  (mg/dL) | TNI  (pg/mL) | Ferritin  (ng/mL) | CPK  (U/L) | TG (mg/dL) | CRP  (mg/dL) | PCT  (ng/mL) |
| --- | --- | --- | --- | --- | --- | --- | --- | --- | --- | --- | --- |
| 1 | 154 | 1602 | 304 | 593 | 934 | 3.1 | 1092 | 53 | 129 | 32.9 | NA |
| 2 | 320 | 1085 | 3934 | 1988 | 633 | 260 | 3605 | 621 | 341 | 22.4 | 3.21 |
| 3 | 95 | 1875 | 1068 | 15129 | 258 | 105 | 383 | 550 | 274 | 4.1 | NA |
| 4 | 327 | 1456 | 367 | 679 | 432 | 14.4 | 143 | 116 | 482 | 20.2 | 6.3 |
| 5 | 131 | 320 | 596 | 1398 | 983 | 459 | 728 | 108 | 137 | 32.9 | 1.14 |
| 6 | 200 | 869 | 459 | 620 | 630 | 2.6 | 1358 | 34 | 154 | 13.3 | 2.34 |
| 7 | 106 | 1473 | 920 | 1790 | 604 | 10.6 | 180 | 99 | 197 | 14 | 0.160 |
| 9 | 296 | 532 | 407 | 611 | 658 | 4.7 | 804 | 407 | 131 | 24.7 | 0.22 |
| 10 | 83 | 652 | 422 | 1549 | NA | 19.9 | 761 | 74 | 148 | 11.9 | 0.27 |
| 11 | 110 | 363 | 306 | 238 | 632 | 8.3 | 547 | 61 | 142 | 16.5 | 0.06 |
| 12 | 111 | 844 | 1071 | 3558 | 724 | 29.4 | 2449 | 536 | 287 | 21.8 | 0.07 |
| 13 | 244 | 567 | 327 | 1015 | 842 | 10.2 | 2739 | 62 | 142 | 36.6 | 1.87 |
| 14 | 90 | 602 | 817 | 4335 | 828 | 205 | 1824 | 215 | 304 | 27 | 3.3 |

*ALC: absolute lymphocyte count, LDH: lactate dehydrogenase, DD: D-dimer, Fg: fibrinogen, TNI: troponin I, CPK: Creatine phosphokinase, TG: triglyceride, CRP: C-reactive protein, PCT: procalcitonin*
